# Computational proteomics analysis of *Taphrina deformans* for the identification of antifungal drug targets and validation with commercial fungicides

**DOI:** 10.3389/fpls.2024.1429890

**Published:** 2024-11-07

**Authors:** Waqar Ahmad, Ziaur Rahman, Haji Khan, Javed Nawab, Hazir Rahman, Muhammad Faisal Siddiqui, Wajeeha Saeed

**Affiliations:** ^1^ Department of Microbiology, Abdul Wali Khan University Mardan, Mardan, Khyber Pakhtunkhwa, Pakistan; ^2^ Centre of Biotechnology and Microbiology, University of Swat, Swat, Khyber Pakhtunkhwa, Pakistan; ^3^ Department of Environmental Sciences, Kohat University of Science and Technology, Kohat, Khyber Pakhtunkhwa, Pakistan; ^4^ Department of Microbiology, Hazara University, Hazara, Khyber Pakhtunkhwa, Pakistan; ^5^ Department of Biology, University of Haripur, Haripur, Khyber Pakhtunkhwa, Pakistan

**Keywords:** *T. deformans*, *Prunus persica*, antifungal targets, fungicides, docking, simulation

## Abstract

*Taphrina deformans* is a plant-pathogenic fungus and a responsible agent for causing peach leaf curl disease. *Taphrina deformans* affects peach fruit production and contributes to global economic losses. Commercial fungicides may provide temporary relief; however, their overuse resulted in adverse environmental consequences as well as led to drug-resistant strains of *T. deformans*. Therefore, the discovery of novel drug targets for the future synthesis of antifungal drugs against *Taphrina deformans* is needed. Here we studied *Taphrina deformans* by computational proteomics approaches. The whole genome and proteome of *T*. *deformans* were subjected to subtractive proteomics, high-throughput virtual screening, and molecular dynamic simulations. We employed subtractive proteomics analysis of 4,659 proteins extracted from UniProtKB database; after filtering out homologous and non-essential proteins, we identified 189 essential ones, including nine that participated in the crucial metabolic pathways of the pathogen. These proteins were categorized as nuclear (*n* = 116), cytoplasmic (*n* = 37), and membrane (*n* = 36). Of those essential proteins, glutamate–cysteine ligase (GCL) emerged as one promising target due to its essential function for glutathione biosynthesis process which facilitates *T. deformans* survival and pathogenicity. To validate GCL as an antifungal target, virtual screening and molecular docking studies with various commercial fungicides were carried out to better characterize GCL as a drug target. The data showed strong binding affinities for polyoxin D, fluoxastrobin, trifloxystrobin, and azoxystrobin within the active site of GCL. Polyoxin D showed a strong affinity when the measured docking score was at -7.34 kcal/mol, while molecular dynamics simulations confirmed stable interactions (three hydrogen bonds, two hydrophobic bonds, and one salt bridge interaction), supporting our findings that GCL represents an excellent target for antifungal drug development efforts. The results showed that GCL, as an innovative target for future fungicide designs to combat *T. deformans infections*, provides an avenue toward creating more effective peach leaf curl disease treatments while mitigating environmental harm caused by its current use.

## 1 Introduction

*Taphrina deformans* (*T. deformans*), a prevalent plant pathogen, infects peach trees, causing the very well-known peach leaf curl disease (PLCD). Peach leaf curl is a globally important disease in which nectarines, peaches, and sometimes other stone fruits’ leaves are affected, such as those of apricots and almonds (Pecknold, 2015b). The fungus *T. deformans* is phylogenetically classified in subphylum Taphrinomycotina, a basal lineage of the phylum Ascomycota that includes fission yeast and members of the genus *Pneumocystis*. The genus *T. deformans* is one of the most extensively studied species because it is the most widespread plant pathogen, infecting the cultivars of nectarine and peach (Cissé et al., 2013). PLCD causes $2.5 to $3 million losses annually in the United States (Chester, 1947). In northern Italy, it can affect 60% to 90% of shoots, representing an important threat to the host tree (Kern and Naef-Roth, 1975). The severity of the disease as well as the resulting economic impact on yield can vary based on the microclimate as well as the different resistance responses of selected cultivars (Rossi et al., 2007).

The fungus induces a fast and spontaneous proliferation of the developing cells at the leaf margins, resulting in a curled, puckered, and blurred shape. The color of the leaves also varies, from variants of yellow and green to brown, purple, and pink. On the surface of the leaves, spores are formed as leaves mature, causing a dusty look (Kurtzman et al., 2011). *T. deformans* enter the leaves’ cells through the stomata (Svetaz et al., 2017). The mycelial growth of fungus in the stomata and interstitial spaces of leaves effects the metabolic activity of the plant (Kolattukudy, 1985). In comparison, the interface between the leaf cell wall and fungus is changed to facilitate fungal nutrition (Bassi et al., 1984). During the infection, changes in the cell host anatomy were also identified (Giordani et al., 2013). When cold and moist conditions are persistent during bud growth, the probability of extreme leaf curl outbreaks is high (Giosuè et al., 2000).

Pathogenicity in *Taphrina deformans*, the causal agent of peach leaf curl, is a multifaceted process that involves a complex interplay of genes and metabolic pathways. These virulence genes belong to cutinase activity, fat metabolism, and other oxidative stresses, favoring the pathogenesis of *T. deformans* in peach plants. The genes enable the fungus to adapt and thrive within its host environment. Research indicates that the synthesis of chitin, a critical component of the fungal cell wall, is essential to maintain cell integrity and facilitate successful infection. Genes that regulate chitin production and modification are therefore pivotal in the pathogenicity of this fungus (Tsai et al., 2014). In terms of fungal management, several fungicides are employed to control *T. deformans*. Remarkably, certified bio-fungicides like polyoxin D target chitin synthase directly compromise the fungus’ ability to maintain its cell wall structure. Additionally, strobilurin fungicides such as fluoxastrobin, trifloxystrobin, and azoxystrobin inhibit the mitochondrial cytochrome bc1 complex, disrupting ATP production and ultimately leading to fungal cell death. These mechanisms highlight the importance of targeting both structural components, like the fungal cell wall, and energy production pathways in the development of effective fungicides against *T. deformans* (Tsai et al., 2014).

The genome of *T. deformans* has been sequenced, revealing approximately 5,735 protein-coding genes, many of which are implicated in plant cell wall degradation, secondary metabolism, and the biosynthesis of plant hormones, all of which contribute to its virulence and pathogenicity. In addition, the presence of drug detoxification enzymes in its genome suggests that *T. deformans* can develop resistance to fungicides, complicating control efforts (Cissé et al., 2013). Knowing the genetic and biochemical pathways involved in the pathogenicity of *T. deformans* is crucial for the development of targeted management strategies and novel fungicides that can effectively mitigate the impact of this significant agricultural pathogen (Maniatis et al., 2024).

Understanding the molecular basis of pathogens that threaten agriculture, like *Taphrina deformans*, and their resistance mechanisms against fungicides is crucial in combatting fungal pathogens. Widely utilized strategies include a single spray in early spring that can effectively combat the disease due to the monocyclic nature of this pathogen (Fitzpatrick, 1934). Spraying fungicides, such as Chlorothalonil, Bordeaux, and copper-based products, is able to prevent PLCD by killing fungal spores, thus preventing the spread of infection (Gogorcena Aoiz et al., 2020; La Torre et al., 2018). The fungicides are sprayed twice a year in spring and autumn; however, fall season is mostly deemed suitable for effective control (Thomidis et al., 2018). The excessive use of fungicides has adverse effects on the environment. Furthermore, fungal pathogens developed resistance against commercial pesticides (Zhang et al., 2015). Currently, the scientific community is trying to discover and develop new drug and drug targets in fungal pathogens. In this study, we have identified novel drug targets in *T. deformans* and characterized them with commercial fungicide using *in silico* methodology.

Furthermore, due to the extensive and long-term use of fungicides, the pathogens develop resistance against registered ones (Zhang et al., 2015). Therefore, the use of new fungicides against novel targets, which can emerge as effective alternatives to the fungicides currently used, is important. Our research is focused on glutamate–cysteine ligase (GCL), an essential enzyme involved in glutathione biosynthesis that plays an essential role in managing oxidative stress—an integral factor of pathogen viability directly influencing its ability to infect and damage the host plant. Traditional fungicides like polyoxin D and azoxystrobin target this essential protein, disrupting essential metabolic pathways. In this study, a more detailed examination of this interaction is being undertaken in order to validate GCL as a potential new drug target, potentially leading to more effective fungicidal strategies.

To identify the drug target, the proteome of *T. deformans* was subjected to subtractive proteome analysis; the non-homologous and essential proteins (uncharacterized) were identified against the *Prunus persica* proteome. Moreover, virtual screening and molecular dynamics (MD) simulations, which are efficient computational biology strategies, were used to identify inhibitory compounds and reaffirm their stability. We identified glutamate–cysteine ligase (GCL) as a target for antifungal drugs by virtual screening, molecular docking simulations, and protein–protein and protein–drug interactions. To our knowledge, this is the first report that shows GCL as a target for future drug development against *T. deformans*. The docking results show strong binding and stability of the GCL active site with polyoxin D, fluoxastrobin, trifloxystrobin, azoxystrobin, vincozolin, and propiconazole. These results validate that GCL could be a novel target to control the growth of *T. deformans* and PLCD.

## 2 Materials and methods

### 2.1 Retrieval and identification of uncharacterized proteins

**Figure 1:**
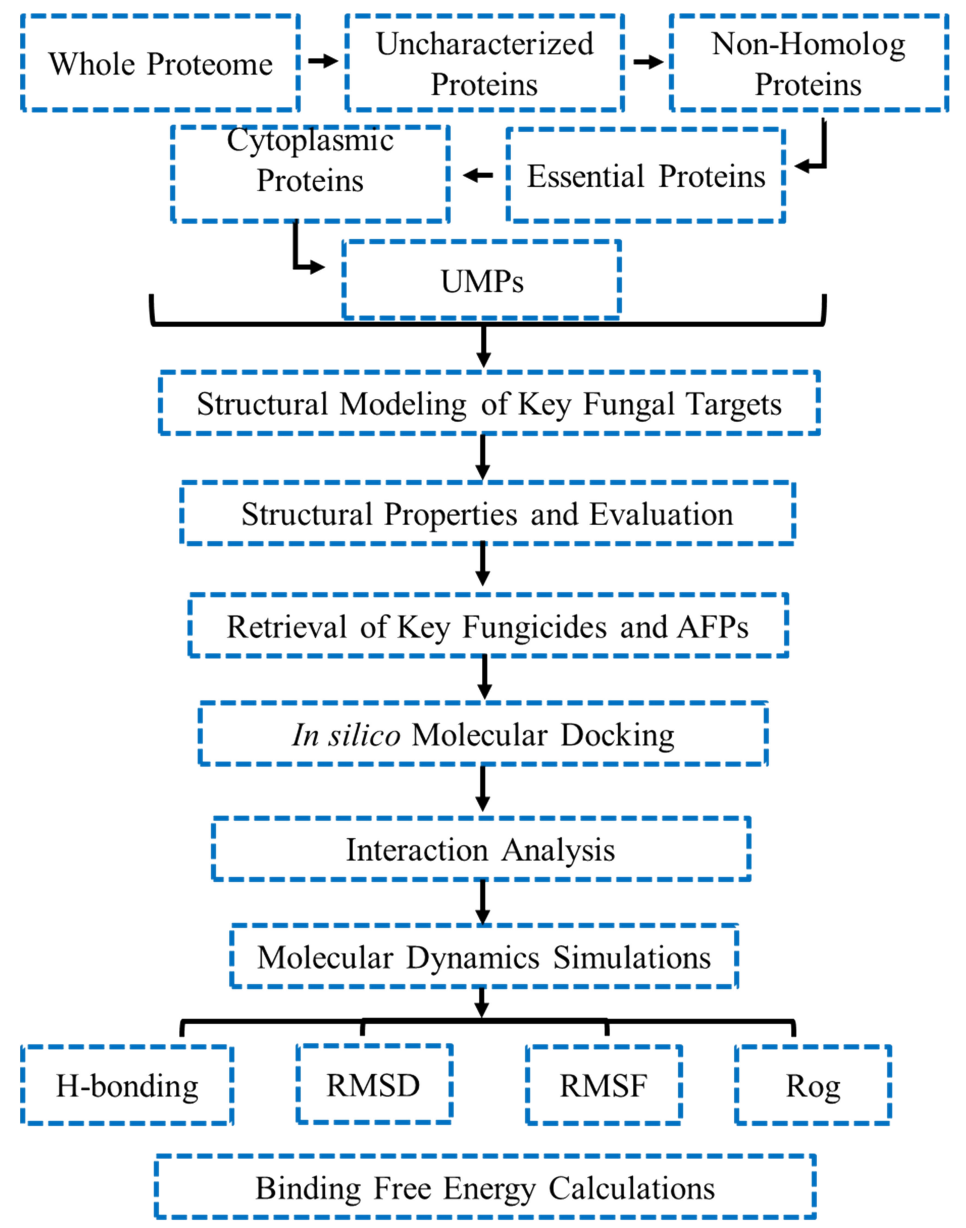
Detailed methodological workflow of the study. UMP, unique metabolic pathways; RMSD, root mean square deviation; RMSF, root mean square fluctuation; ROG, radius of gyration.

The whole protein contents of *T. deformans* were downloaded from the UniprotKB database (https://www.uniprot.org/) that comprised of 4,659 protein sequences (Consortium, 2019). The uncharacterized proteins of *T. deformans* were identified for further analysis. The graphical workflow of methodology is illustrated in **Figure 1**. A list of the tools used in the study is provided in **Supplementary Table S1**.

### 2.2 Identification of paralogous sequences

The selected uncharacterized proteins were subjected to the Cluster Database at High Identity with Tolerance CD-HIT server, a cluster database at high identity with tolerance (http://weizhong-lab.ucsd.edu/cdhit-web-server/cgi-bin/index.cgi) for the identification and subsequent removal of paralogous proteins. Paralogous proteins are duplicates that arise during evolution (Li and Godzik, 2006). Redundant proteins that are duplicated or repeated proteins that are irrelevant were considered as targets for a potential fungicide. Generally referred to as paralogous, these proteins have no specific target-based details, so it was convenient to exclude them prior to analysis (Ahmad et al., 2022). For the CD-HIT analysis, a threshold value of 80% sequence identity was deemed to be paralogous in nature (Li and Godzik et al., 2006).

### 2.3 Non−homology analysis against *Prunus persica*

The non-paralogous proteins were searched for non-homology against the whole proteome of *Prunus persica*. NCBI-BLASTp search (https://blast.ncbi.nlm.nih.gov/Blast.cgi?PAGE=Proteins) against the proteome of *Prunus persica* was carried out with an e-value 0.0001 and bit score threshold >100 for the identification of proteins that are non-homologous to the host (Gul et al., 2020). It is an essential step in subtractive proteomics analysis that ensures that the fungicide will not damage the host since the target protein has no similarity to the host proteins.

### 2.4 Identification of essential proteins

The non-homologs were subjected to the identification of essential proteins that play a key role in the survival of the pathogen by searching the Database of Essential genes (http://www.essentialgene.org/). The e-value was set to 0.0001 and bit score >100 to classify the essential proteins (Zhang et al., 2004).

### 2.5 Sub−cellular localization

The non-homologous and essential uncharacterized proteins are divided into two categories depending on protein localization: cytoplasmic proteins that can better serve as candidates for fungicide target and non-cytoplasmic proteins. Localization of the essential proteins was performed by using CELLO2GO server (http://cello.life.nctu.edu.tw/cello2go/) (Yu et al., 2006). CELLO2GO employs the BLAST algorithm to find homologous sequences, which are GO annotations in a search database used in the work modified from UniProtKB/SwissProt. The cytoplasmic proteins were set forth for further analysis. The confidence score for the target proteins was set from 0 to 1, where 0 indicates low clustering and 1 corresponds to the likelihood of presence in the desired cluster.

### 2.6 Pathway analysis

**Figure 2:**
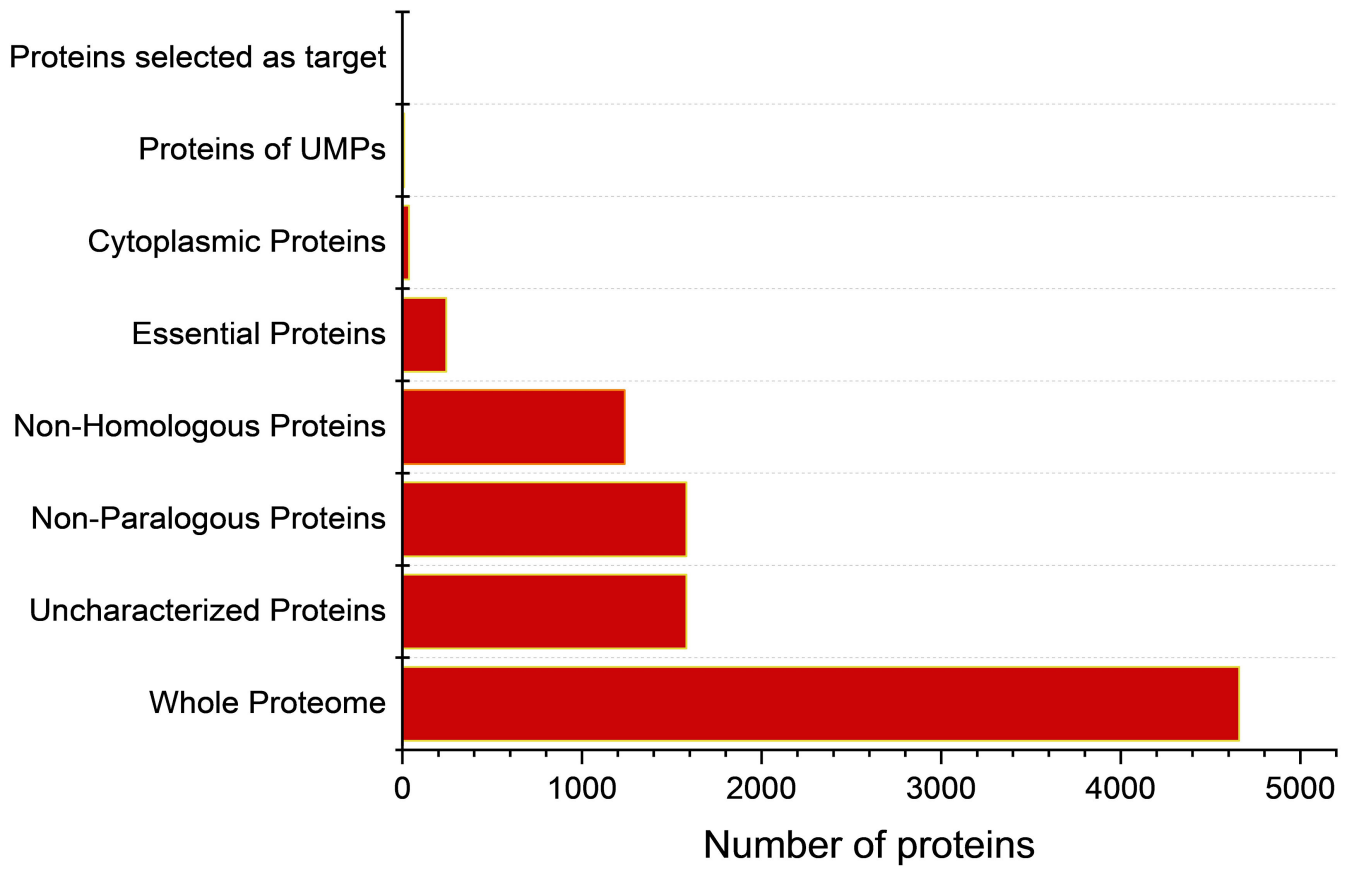
Subtractive proteomics filtering for novel fungicide target prioritization.

Pathway analysis was carried out for selected cytoplasmic proteins using KEGG database. To identify the metabolic pathway of non-homologous proteins in *T. deformans*, Kyoto Encyclopedia of Genes and Genomes (KEGG) or Genome Database (https://www.genome.jp/kegg/kaas/) was used (Moriya et al., 2007). Similarly, a metabolic pathway study of the host was also conducted. A manual comparison was carried out using the KAAS server for qualitative insights into the similarity of pathogen–host metabolic pathways. Finally, proteins have been distinguished according to their corresponding role in the metabolic pathways present only in the pathogen and absent in the host; the workflow of this study is shown in **Figure 2**.

### 2.7 Homology modeling

The 3D model is not accessible within the Protein Data Bank (PDB) for the hypothetical protein due to the lack of clarity in the descriptions of conserved areas. Thus, homology modeling was used to construct this 3D model. Phyre2 server was utilized for homology modeling of the new target protein for fungicides (Kelley et al., 2015). The concept of homology modeling is to create 3D models of protein structures through referring to the templates of relatives of the same family. The Phyre2 server is based on the creation of 3D models of proteins that evaluate proteins based on template models of particular protein families, the structures of which nuclear magnetic resonance (NMR) spectroscopy or X-ray is able to solve in a laboratory environment. Phyre2 employed sophisticated remote homology detection techniques to create 3D models.

#### 2.7.1 Tertiary structure prediction and validation of glutamate–cysteine ligase

The primary amino acid sequence of glutamate–cysteine ligase (GCL), consisting of 647 amino acids, was subjected to tertiary structure prediction using the Phyre2 web-based modeling server. The sequence was input into Phyre2, and structural modeling was carried out by utilizing multiple templates (*Saccharomyces cerevisiae*, *Salmonella Typhimurium*, and *Escherichia coli*). The modeled structure was generated with 90% confidence level, indicating reliable structural accuracy. The COFACTOR server was used to predict the active site of the modeled protein according to Kelley et al. (2015).

### 2.8 Validation of protein model

The Phyre2-constructed model was subjected to 3D protein model validation. ERRAT and Ramachandran plot evaluation are widely used for structural assessment. Evaluation of the protein models was carried out to determine if the model is correctly configured (Khan et al., 2019, 2022). In general, structure validations evaluate the acceptable and not acceptable conformations of the models. The validation of most structures seeks to track the R-value and resolution as a greater resolution indicates a higher precision of molecular structures.

ProSA (Protein Structure Analysis) is a widely used method comprising a diverse user base and is mainly used to analyze and validate the predicted protein mode (Wiederstein and Sippl, 2007). ProSA specifies the analysis of X-ray and NMR spectroscopy. The errors in protein structure were recognized by the server to identify the regions and promote the method of interpretation. Procheck carries out protein model validation through the Ramachandran plot (Laskowski et al., 1993). The analysis was carried out to classify the amino acids of the protein models within the preferred and disallowed regions. The high-quality model should show a cumulative score of more than 90% for amino acids in the centralized and approved regions. The ERRAT plot was utilized to verify the protein models by producing the “overall performance factor” of the non-bonded atomic interactions (Dym et al., 2012). This step evaluates the protein 3D model generated utilizing homology modeling as well as the higher score model representing high-quality models.

### 2.9 High-throughput virtual screening

Fungicides were virtually screened using AutoDock Vina against the binding sites of a protein. The identified binding sites have features about the degree of exposure, enclosure, hydrogen bonding, size, tightness, hydrophobicity, linking site points, and hydrophilic nature. Firstly, the complete database was screened using AutoDock Vina, and then finally, to confirm the final hits for the best scoring compounds, induced-fit docking (IFD) was done through 64 exhaustiveness. For IFD AutoDockFR, AutoDock with flexible receptors (ADFR) was done (Ravindranath et al., 2015). It utilizes the AutoDock4 scoring characteristic to lower the internal electricity of the receptor. It also handles the side chain of the receptor conformational optimization of up to 14 unique facet chains that enhance the docking rate. AutoDockFR has better accuracy over AutoDock Vina in skip-validation docking, and the speed of docking is much more efficient.

### 2.10 Molecular dynamics simulation of protein–ligand complexes

In order to perform all-atoms MD simulation, Amber18 package was used for top hits from in-house fungicide database (Case et al., 2005). The topologies of drug small molecules were predicted using an antechamber module, while for complex simulations, the Amber general force field (GAFF) and ff14SB force fields were employed. A TIP3P container of water and Na^+^ counter ions have been used to solvate and neutralize each device eventually. The energy minimization of systems was carried out in two stages, followed by heating and equilibration. The Particle Mesh Ewald (PME) algorithm is used to quantify lengthy-range electrostatic interactions (Price and Brooks III, 2004). A 1.4-nm cutoff value was set for Van der Waals interactions as well as for columbic interactions of short range. Langevin thermostat was employed to temperature constant at 300 K, whereas for pressure control, Berendsen barostat was considered. A time step of 2fs and total simulation time of 50 ns for each complex were performed. The dynamics, stability, and other features of the ligand–protein complexes were evaluated by using CPPTRAJ and PTRAJ within AMBER software suite (Roe and Cheatham III, 2013).

### 2.11 Calculations of binding free energy

For all of the protein–ligand complexes, the binding free energy was calculated using the MMPBSA.PY script (Python-based script used to perform molecular mechanics Poisson–Boltzmann surface area) by considering 500 snapshots. This method is widely used to estimate the total binding energy (TBE) of various ligands, as referenced in several studies. Binding free energy is computed using the equations below (Hou et al., 2012; Miller III et al., 2012; Sun et al., 2014b; Chen et al., 2016). Different studies broadly use this unfastened energy calculation approach to estimate the TBE of various ligands (Wang et al., 2018).


(1) $${\text{ΔG}}_{\text{bind}}{\text{=ΔG}}_{\text{complex}}-\left[{\text{ΔG}}_{\text{receptor}}{\text{+ΔG}}_{\text{ligand}}\right]$$

ΔG_bind_: overall free binding energy—the energy change associated with the binding of a ligand to a receptor,

ΔG_complex_: free energy of the complex or the energy state of the receptor–ligand complex

ΔG_receptor_: free energy of the receptor or the energy state of the receptor alone (without the ligand)

ΔG_ligand_: free energy of the ligand or the energy state of the ligand alone (without the receptor)

Equation 2 was used to calculate the specific energy term contribution to the total free energy:


(2) $${\text{G=G}}_{\text{bond}}{\text{+G}}_{\text{ele}}{\text{+G}}_{\text{vdW}}{\text{+G}}_{\text{pol}}{\text{+G}}_{\text{npol}}$$

G: total free energy

G_bond_: bonding energy (or bond stretching energy)

G_ele_: electrostatic energy (or Coulomb energy)—energy due to electrostatic interactions between charged particles

G_vdW_: van der Waals energy (or dispersion energy)

G_pol_: polarization energy—energy associated with the polarization of molecules (e.g., induced dipoles)

G_npol_: non-polar energy (or hydrophobic energy)

This equation accounts for bonded interactions, electrostatic energy, van der Waals forces, polar solvation energy, and non-polar solvation energy, thus providing a comprehensive view of the binding interactions involved.

## 3 Results

### 3.1 Retrieval and identification of uncharacterized proteins

The complete proteome of *Taphrina deformans* (Proteome ID: UP000013776) comprised 4,659 proteins—1,581 of which were identified as uncharacterized proteins. The molecular functions of more than 30% of proteins remain unknown in most organisms; such proteins are referred to as hypothetical or uncharacterized proteins. The functional characterization of such proteins can help us to consider their functions in various metabolic pathways essential for the survival of the pathogen and to classify previously novel fungicide targets in plant pathogens (Shahid et al., 2016). There are several bioinformatics services available for the functional classification of uncharacterized proteins, like databases and software. The functions of uncharacterized proteins in different microbial pathogens have been successfully annotated using these resources, including *Borrelia burgdorferi* (Khan et al., 2016), *Chlamydia trachomatis* (Naqvi et al., 2017)], *Haemophilus influenza* (Shahbaaz et al., 2013), and *Vibrio cholerae* (Islam et al., 2015), and can be utilized to identify potential fungicide candidates in *T. deformans.* The subtractive proteomics analysis is graphically illustrated in **Figure 2**.

### 3.2 Identification of non-paralogous proteins

By importing the FASTA format of uncharacterized proteins into the CD-HIT server, the non-paralogous proteins were identified. This server implements greedy incremental algorithms to remove redundancy and cluster protein sequences. The proteins were sorted depending on a chosen sequence identity cutoff depending on the requirement. The sequence identity of 80% was chosen as a cutoff to preserve a rigid parameter in this analysis. The entire proteome was clustered, and proteins with 80% identity were identified and analyzed as paralogous. After the CD-HIT suite study, no duplicates or paralogs with more than 80% identity were found (**Figure 2**). The proteins were set forth for identification and subsequent removal of proteins similar to the host.

### 3.3 Non−homology analysis against *Prunus persica*

Uncharacterized proteins were subjected to NCBI-BLASTp search against *Prunus persica* proteome, with a threshold e-value of 0.0001, to identify the non-homologous sequence. The homologous sequences were identified and ultimately excluded from the study. Only those sequences specific to the pathogen and can be regarded as potential fungicide targets were selected after excluding the homologous sequences. In this step, 1,262 uncharacterized non-homologous proteins were identified (**Figure 2**).

### 3.4 Essential proteins for *T. deformans* survival

The non-homologous proteins of *T. deformans* against plant proteome was further treated for the identification of essential genes in *T. deformans*. From the non-homologous sequences, essential proteins were separated with the Blastp technique against the eukaryotic set of the Database of Essential Gene (DEG). We identified a total of *n* = 244 critical proteins (**Figure 2**). A gene product’s practical contribution to an organism’s survival and pathogenicity determines how crucial it is. The output of the sequence from the last phase was locally aligned against the Database of Essential Gene in order to determine essentiality (van Vlijmen et al., 2017). Protein alignments linked to an anticipated value of less than 0.0001 were deemed to be more significant hits. Crucial proteins are essential to pathogen survival and could be viewed as a first step toward developing new fungicide targets.

### 3.5 Subcellular localization

**Figure 3:**
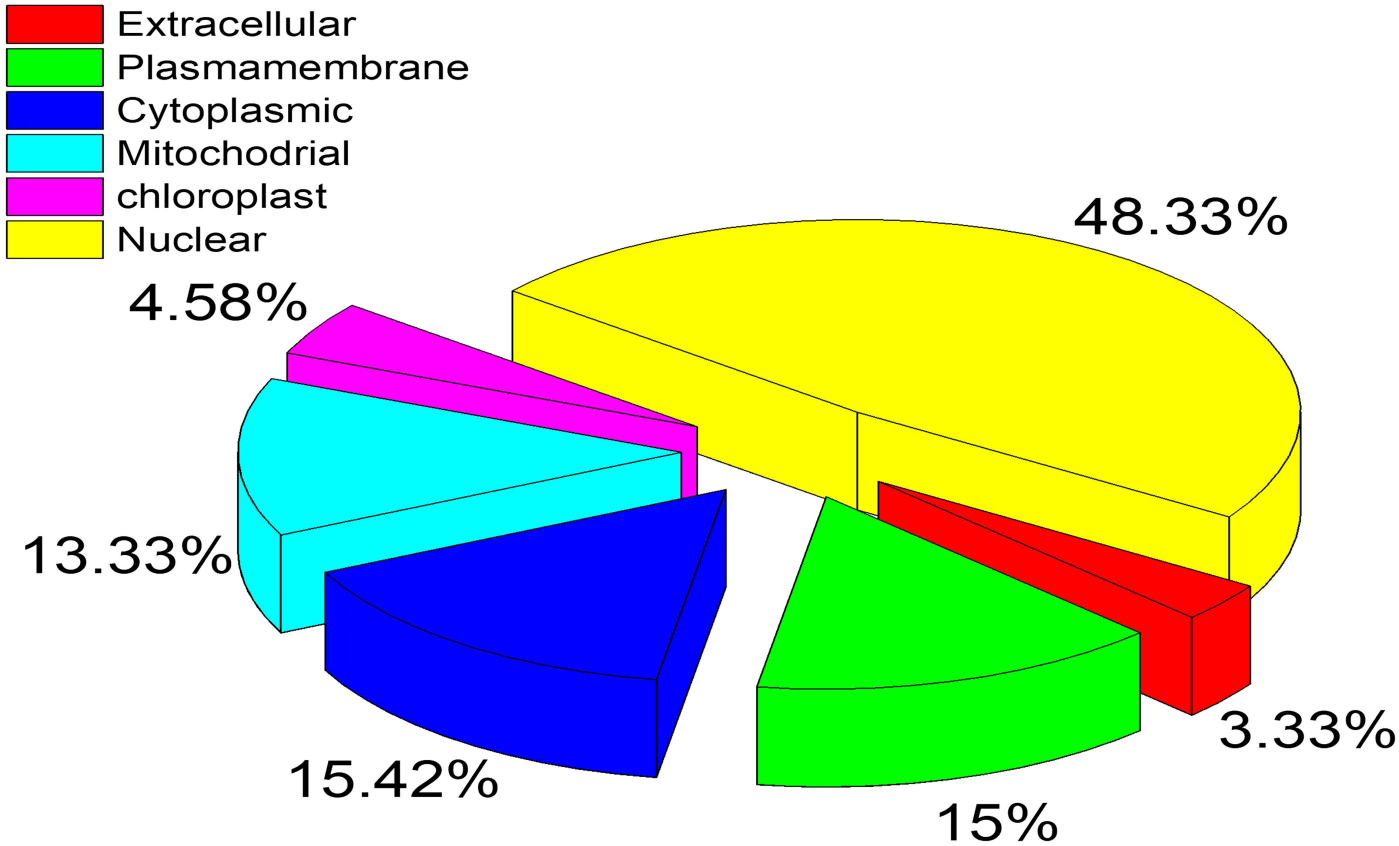
Subcellular location prediction of essential proteins by CELLO2GO server. The respective location is represented by a different color with the percentage of proteins.

The subcellular locations of these proteins were identified by utilizing the CELLO2GO server. The overall prediction revealed *n* = 116 proteins in the nuclear region, 37 proteins in the cytoplasm, and 36 proteins in the plasma membrane. Cytoplasmic proteins have a strong possibility of being a suitable fungicide target. The complete results of the CELLO2GO prediction are provided in the pie chart (**Figure 3**).

### 3.6 Unique pathway analysis

The 13 uncharacterized proteins that are essential for the survival of *T. deformans* and located in the cytoplasm were subjected to pathway analysis using the KAAS tool available in the KEGG database. These proteins were classified into distinctive pathways according to their functions and significance in unique metabolic pathways. Not only do KAAS outputs provide convenient metabolic routes but they also have remarkable informational features, such as KO list projects and opportunity pathways of enzymes and Enzyme Commission (EC) numbers. The KO list of *T. deformans* uncharacterized proteins was compared to host metabolic pathways that revealed nine proteins having key roles in metabolic pathways unique to the pathogen. The unique metabolic pathways (UMPs) are shown in **Table 1**. The uncharacterized protein glutamate–cysteine ligase (Uniprot ID: R4XJV2) was identified as the most suitable fungicide target. It is involved in the cysteine and methionine metabolism, glutathione metabolism, and ferroptosis pathways. The target protein is involved in step one of the subpathway that synthesizes glutathione from L-cysteine and L-glutamate (Fahey and Sundquist, 1991).

**Table 1:** Identified unique metabolic pathways (UMPs) in the *T. deformans* essential proteins located in the cytoplasm.

Uniprot ID	KO ID	UMPs	Pathway
R4XM80	K16186	mTOR signaling pathway	ko04150
R4XDE1	K11559	Chromosome and associated proteins	ko03036
R4XAL9	K10416	Phagosome	ko04145
R4XFC4	K01078	Thiamine metabolism	ko00730
		Riboflavin metabolism	ko00740
R4XAA5	K13238	Fatty acid degradation	ko00071
R4XJV2	K11204	Cysteine and methionine metabolism	ko00270
		Glutathione metabolism	ko00480
		Ferroptosis	ko04216
R4XL75	K03068	mTOR signaling pathway	ko04150
R4XEZ0	K15206	Transcription machinery	ko03021
R4XD17	K15620	Transcription machinery	ko03021

### 3.7 Tertiary structure prediction and validation of target protein

**Figure 4:**
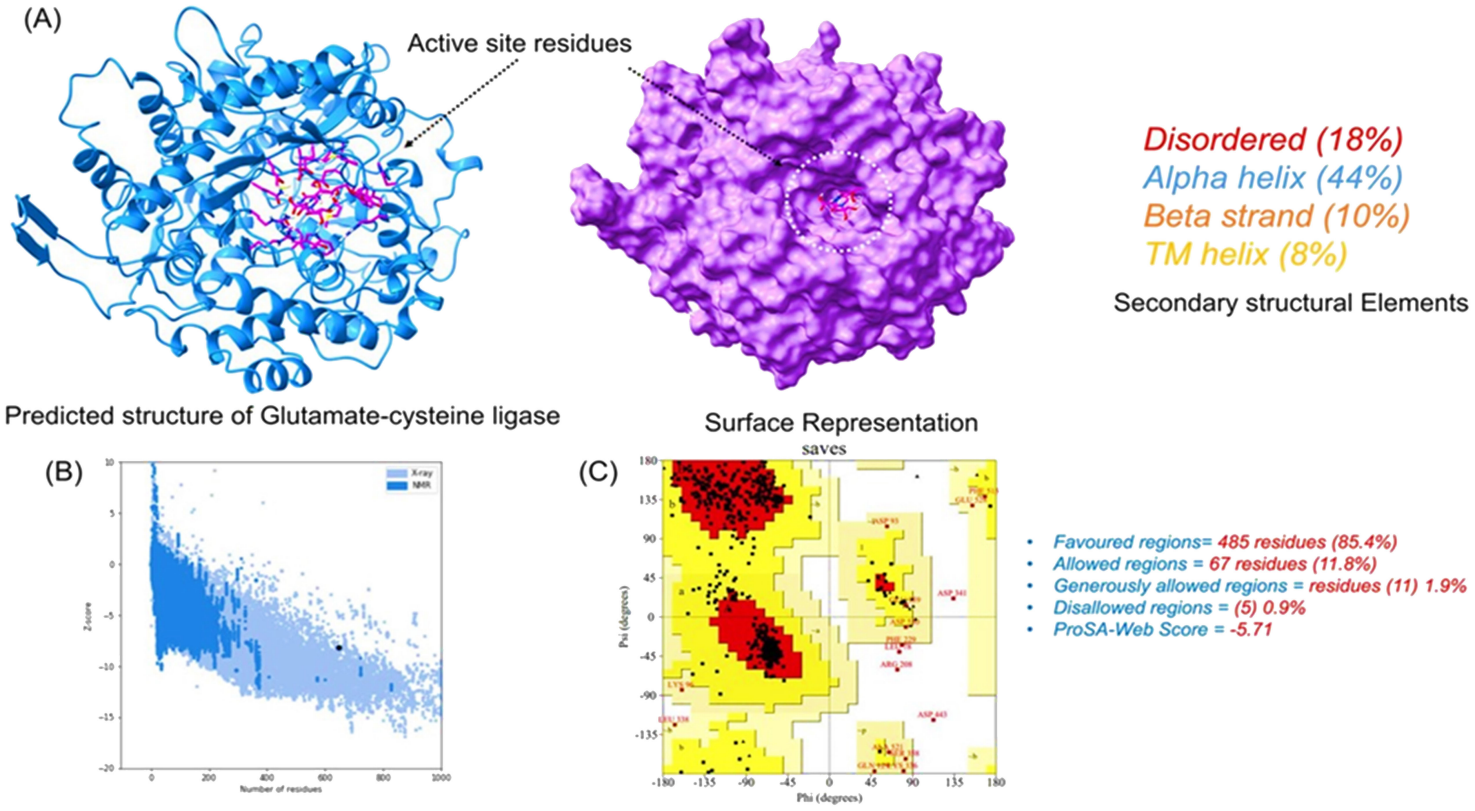
Structural modeling and evaluation of the predicted 3D structure. **(A)** 3D modeled structure with the active site residues. **(B)** ProSA-Web results (*Z* = -5.71), while the Ramachandran plot obtained from SAVES server is given in **(C)**.

The primary amino acid sequence of glutamate–cysteine ligase was processed for 3D structural modeling using Phyre2 web modeling server. A 647-amino-acid-long sequence was modeled with 90% confidence by using multiple templates. The query sequence shares 97% sequence identity with glutamate–cysteine ligase from *Saccharomyces cerevisiae* and 21% sequence identity with glutamine synthetase of *Salmonella Typhimurium* and *Escherichia coli*. The visual analysis shows that the structure has proper folding with uniformly interspersed secondary structural elements. The structure possesses 44% alpha-helices, 10% beta-sheets, and 18% disordered and 8% trans-membrane helices. The protein’s active site was identified using COFACTOR server and revealed residues Glu50, Glu110, Tyr111, His195, Gln272, Ser427, Trp430, Arg434, and Arg451 as active sites. The modeled 3D structure and the binding site residues are shown in **Figure 4A** as a cartoon and surface representation. Evaluation of the 3D structure was performed using various algorithms, which revealed that most of the residues lie in the favored region while only five residues are in the disallowed region. The ProSA-web (interactive web service for the recognition of errors in the 3D structures of proteins) further confirmed that the Z-score of the modeled structure is -5.71, which reflects the best quality of the 3D structure. Overall, the structural evaluation shows that the structure could be used for further analysis—the evaluation results obtained from different servers as shown in **Figures 4B, C**.

### 3.8 Identification of fungicides as control agents against *T. deformans*

A computational virtual screening algorithm was employed using PyRx to screen the 31 selected fungicides against the active site of glutamate–cysteine ligase. Residues Glu50, Glu110, Tyr111, His195, Gln272, Ser427, Trp430, Arg434, and Arg451 were confirmed as active site residues that can bind to fungicides. The initial screening results revealed the range of docking from -7.34 to -2.38 kcal/mol. Fungicides with this score greater than -6.0 were subjected to a second round of screening using the induced-fit docking approach, which owns the advantage of efficient conformational optimization for better binding. In the first screening, fungicides, i.e., polyoxin D zinc salt, with this score (-7.34 kcal/mol) were identified as the best fungicide. Among the other best hits, fluoxastrobin (docking score = -6.81 kcal/mol), trifloxystrobin (docking score = -6.49 kcal/mol), azoxystrobin (docking score = -6.37 kcal/mol), pyraclostrobin (docking score = -6.31 kcal/mol), vinclozolin (docking score = -6.18 kcal/mol), and propiconazole (docking score = -6.03 kcal/mol) were identified as the best hits which could potentially inhibit the peach fungus *T. deformans* by targeting the glutamate–cysteine ligase protein. The docking results along with the names, PubChem IDs, first screening, and induced fit docking scores of the top hits are given in **Table 2**.

**Table 2:** Fungicide names, their PubChem ID, virtual screening docking scores, and the induced-fit docking (IFD) scores of top hits.

Fungicide name	PubChem ID	Rigid docking score	IFD score
**Polyoxin D zinc salt**	CID72476	-7.34	-7.88
**Fluoxastrobin**	CID11048796	-6.81	-7.7
**Trifloxystrobin**	CID1164966	-6.49	-7.63
**Azoxystrobin**	CID3034285	-6.37	-7.59
**Pyraclostrobin**	CID6422843	-6.31	-7.47
**Vincozolin**	CID39676	-6.18	-6.54
**Propiconazole**	CID43234	-6.03	-6.03

**Figure 5:**
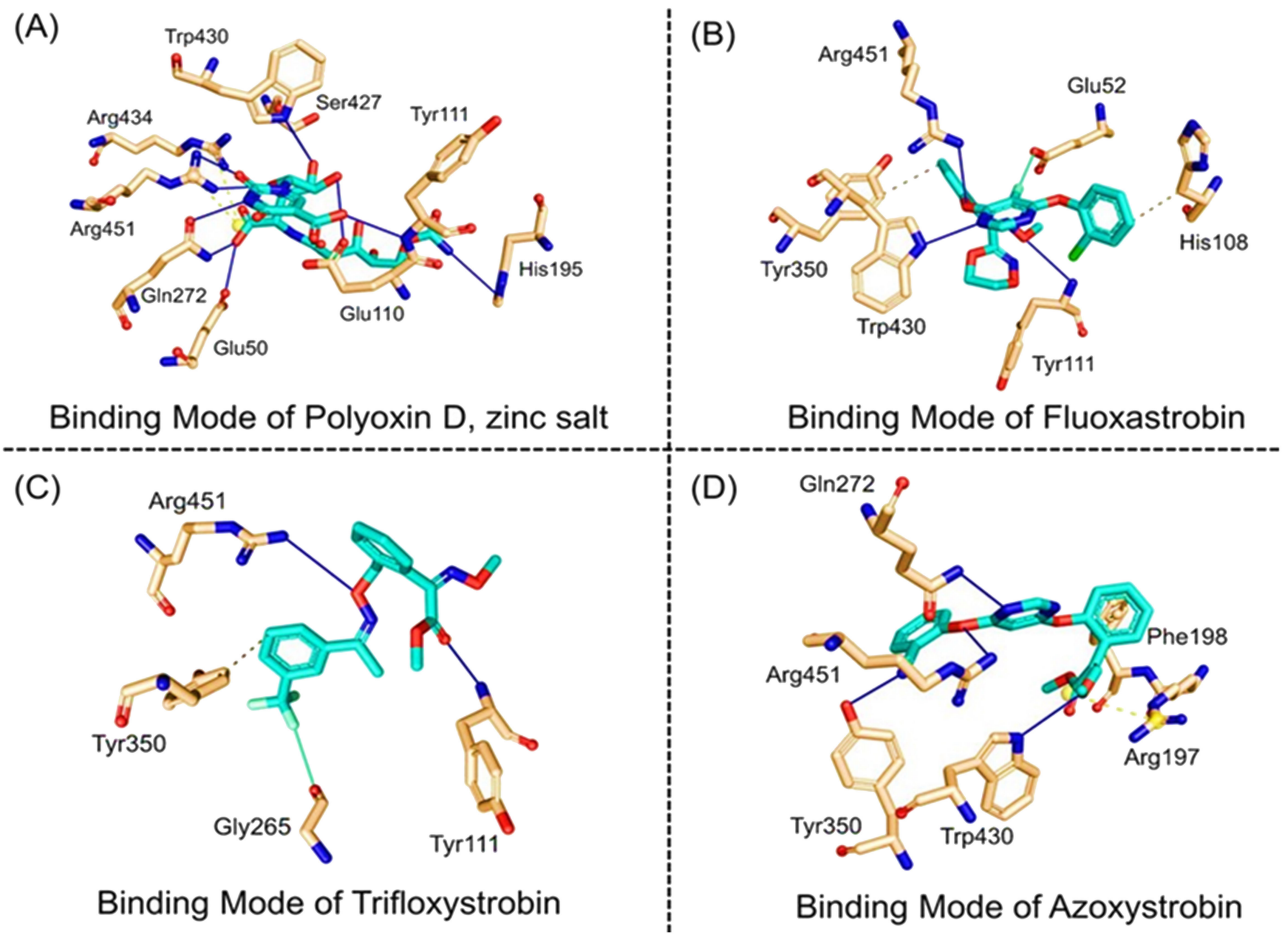
Docking representation of the top hits selected from induced-fit docking (IFD) protocol. **(A)** 3D interaction pattern of polyoxin D zinc salt, **(B)** 3D interaction pattern of fluoxastrobin, **(C)** 3D interaction pattern of trifloxystrobin, and **(D)** 3D interaction pattern of azoxystrobin.

Next, the top hits were again subjected using the IFD protocol to select strong control agents for *T. deformans* infection. The docking score range for the IFD protocol was -7.88 to -6.08 kcal/mol (**Table 2**). Fungicides in the IFD protocol with docking scores greater than -7.50 kcal/mol were selected as the best hits for interaction evaluation and structural–dynamic feature estimation. Among the top hits, polyoxin D zinc salt (with docking score = -7.88 kcal/mol) formed 10 hydrogen bonds, and 23 hydrophobic interactions were reported. Only two hydrophobic interactions were formed by the two arginine active site residues at positions 434 and 451. In the case of the hydrogen bonds, Glu50, His195, Tyr111, His195, Gln272, Ser427, Trp430, Arg434, and Arg451 amino acids were involved (**Figure 5A**). Unlike the polyoxin D zinc salt, fluoxastrobin established only three hydrogen bonds and two hydrophobic bonds, and a single salt bridge was observed. The interaction pattern revealed that Tyr111, Trp430, and Arg451 are involved in hydrogen bonding interaction, His108 and Tyr350 formed a hydrophobic interaction, and Glu52 established the only salt bridge (**Figure 5B**).

Similarly, the only two hydrogen bonds in the trifloxystrobin complex (with docking score = -7.63 kcal/mol) were formed by Tyr111 and Arg451. Only a single hydrophobic interaction with Tyr350 and a salt bridge by Gly265 were observed (**Figure 5C**). The azoxystrobin complex formed four hydrogen interactions, and one hydrophobic interaction with Arg197 was detected (**Figure 5D**). In conclusion, the results show that the shortlisted fungicides robustly interact with the fungus target and could potentially inhibit the growth of *T. deformans* in the field. This pattern of the top hits is shown in **Figure 5**, and the 2D structures of fungicides, PubChem IDs, hydrogen bonding residues, salt bridges, and hydrophobic interactions are shown in **Table 3**.

**Table 3:** 2D structure representation of fungicides, PubChem IDs, hydrogen bonds, salt bridges, and hydrophobic interactions.

2D structure	PubChem ID	H-bonds	Salt bridges	Hydrophobic interactions
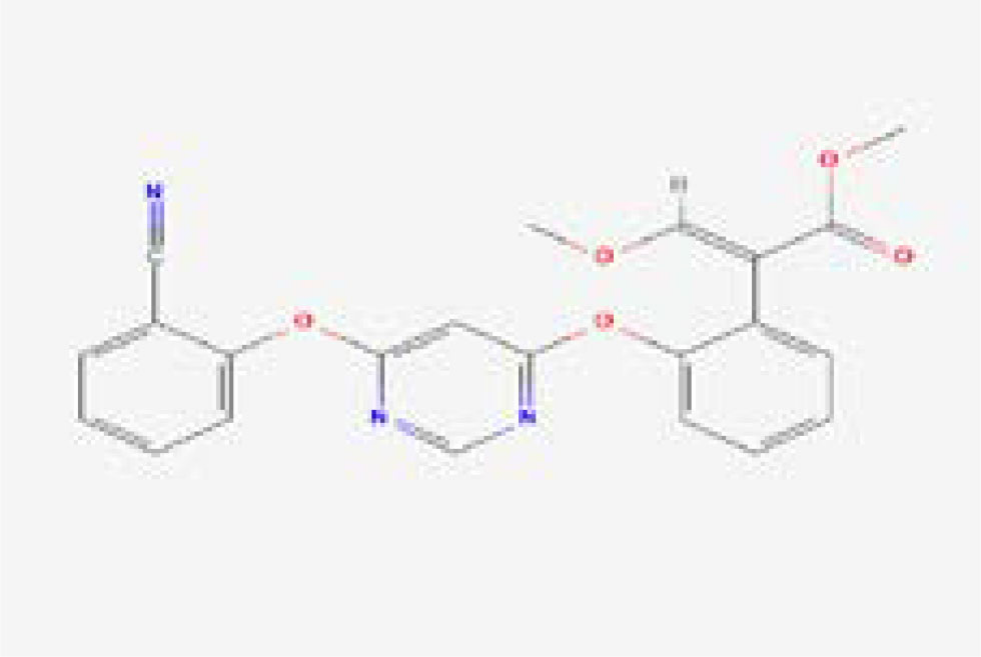	CID72476 Polyoxin D zinc salt	Glu50, His195, Tyr111, His195, Gln272, Ser427, Trp430, Arg434, Arg451	–	Arg434 and Arg451
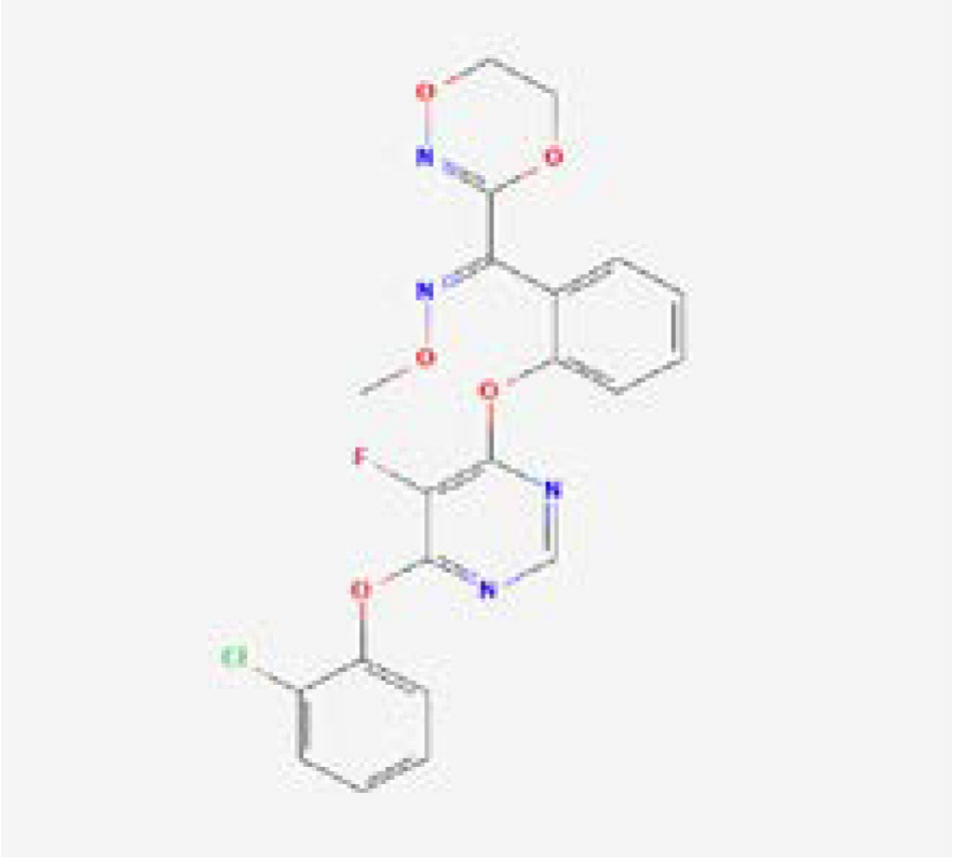	CID11048796 Fluoxastrobin	Tyr111, Trp430, and Arg451	Glu52	His108 and Tyr350
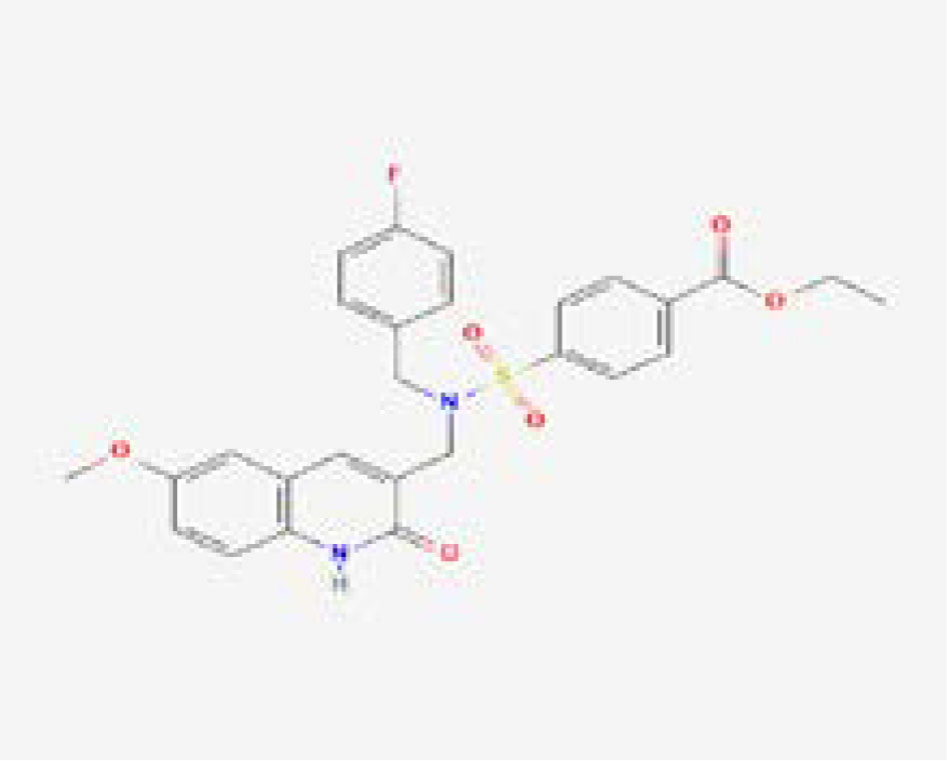	CID1164966 Trifloxystrobin	Tyr111 and Arg451	Gly265	Tyr350
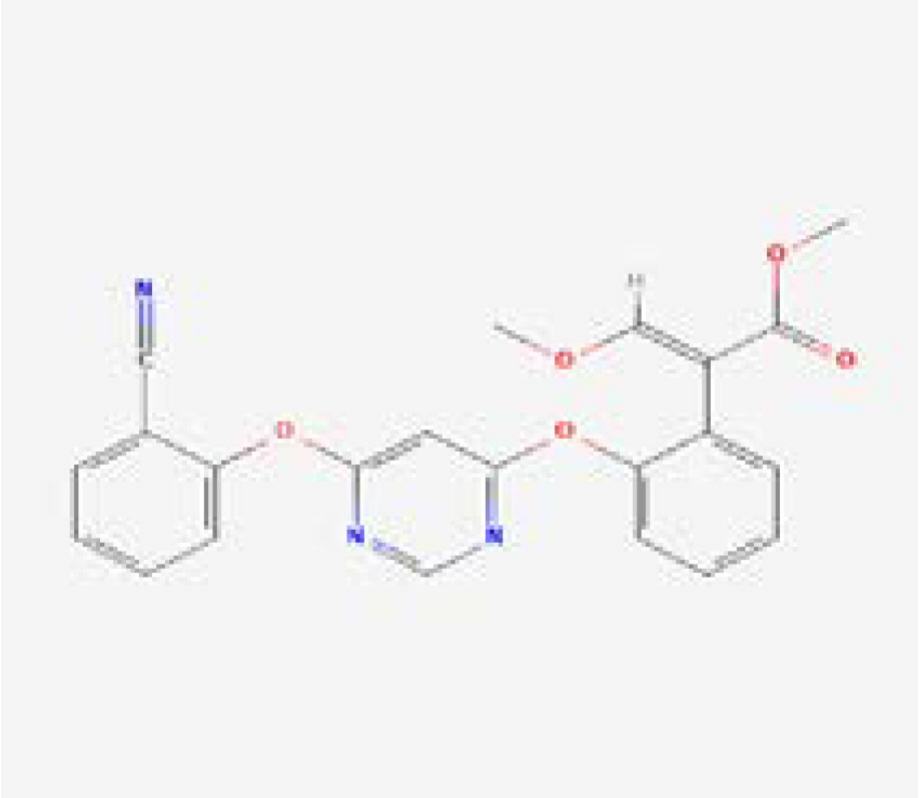	CID3034285 Azoxystrobin	Gln272, Trp430, Arg451, and Arg451	–	Arg197

### 3.9 Dynamic stability estimation of the top hits (fungicides)

**Figure 6:**
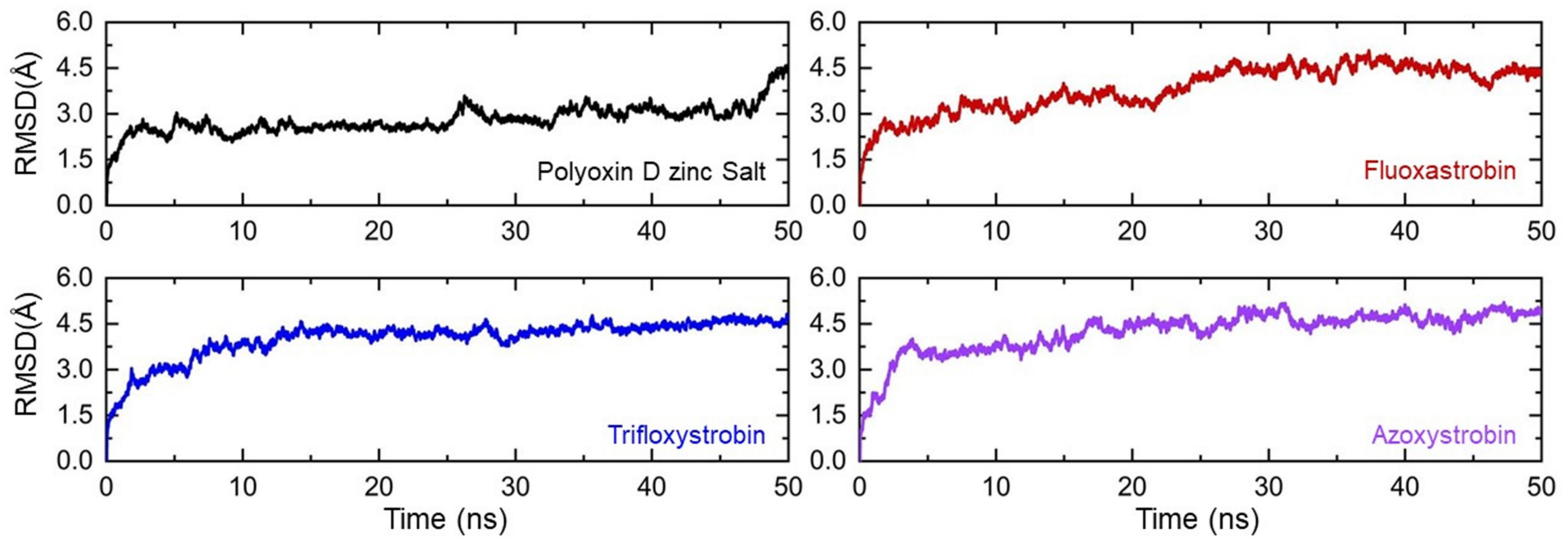
Thermodynamic stability of the top hits calculated as root mean square deviation. Each complex of polyoxin D zinc salt, fluoxastrobin, trifloxystrobin, and azoxystrobin is represented with a different color, respectively.

Two-step fungicide screening approaches shortlisted fungicides, polyoxin D zinc salt, fluoxastrobin, trifloxystrobin, and azoxystrobin as the best hits. The complexes of these top hits were subjected to all-atom molecular dynamics simulation to estimate the thermodynamic stability, residual flexibility, structural compactness, hydrogen bond count, and computations of free energy. Every complex’s thermodynamic stability was determined using the root mean square deviation (RMSD) as a function of time. The RMSDs for each complex demonstrate that throughout the 50ns simulation period, every complex remained stable as shown in **Figure 5**. No significant structural perturbation was observed in any complex. In the polyoxin D zinc salt complex case, the RMSD reached equilibrium at 3 ns, while a slight deviation between 25 and 30 ns was observed. No deviation from the mean structure was observed during the simulation. The average RMSD for polyoxin D zinc salt was observed to be 2.0 Å. On the other hand, the fluoxastrobin complex was also observed to be very stable, but the average RMSD remained higher than the polyoxin D zinc salt complex. Comparatively, fluoxastrobin remained more stable and reached equilibrium at 2 ns (**Figure 6**). Similarly, the trifloxystrobin complex also remained stable, and no structural perturbation was observed, but the RMSD value gradually increased over the simulation time. The average RMSD was observed to be 3.0 Å. Furthermore, the RMSD pattern of azoxystrobin and trifloxystrobin, respectively, was observed to be alike. A gradual increase over the simulation time was recorded in both cases; however, a slightly higher average RMSF for azoxystrobin was observed. Consequently, these results show that all of the fungicides stably remained bound in the binding cavity of GCL and thus possess stronger inhibitory properties. The RMSD graphs of each complex are shown in **Figure 6**.

### 3.10 Structural compactness evaluation

**Figure 7:**
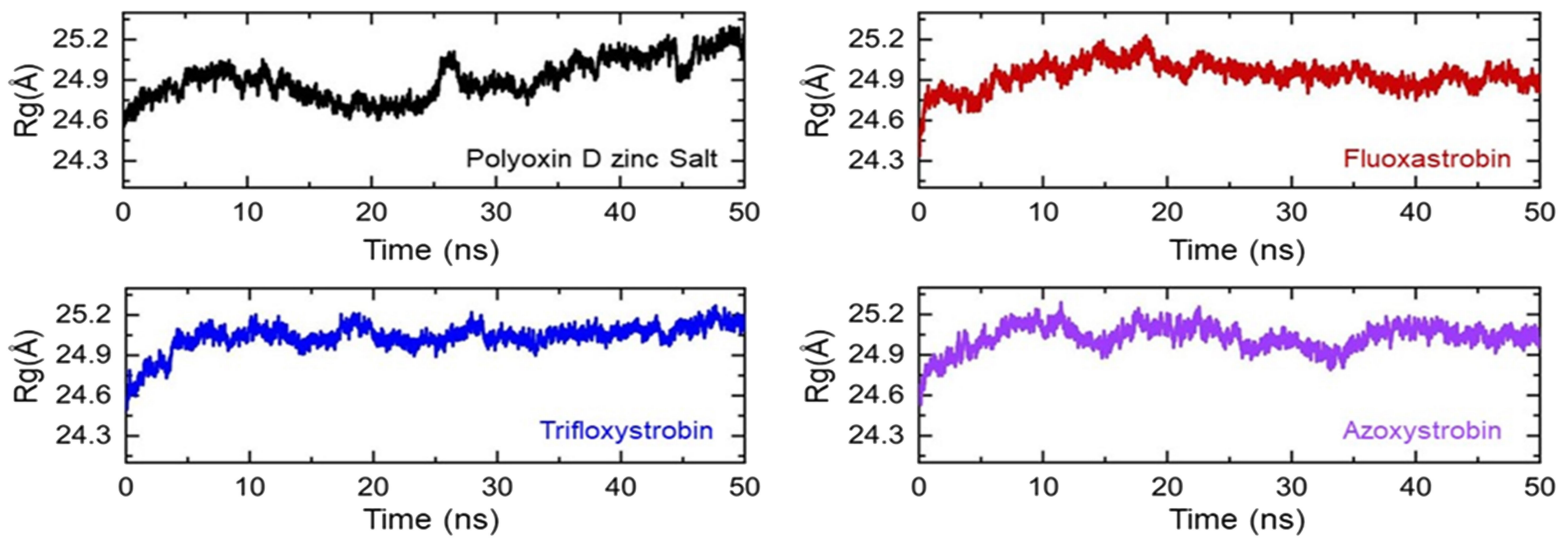
Structural compactness of the top hits calculated as Rg/Rog (radius of gyration). Each complex of polyoxin D zinc salt, fluoxastrobin, trifloxystrobin, and azoxystrobin is represented with a different color, respectively.

The compactness of each fungicide-bound complex was then estimated as its radius of gyration (Rg), and this was assessed to identify any binding or unbinding events that occurred during the simulation. All of the fungicides and GCL protein complexes possess a dynamically compact topology, and no significant variations were observed except in the polyoxin D zinc salt complex (**Figure 7**). The average Rg value for polyoxin D zinc salt was observed to be 24.80 Å.

During the first 25 ns, no significant deviation was observed; then, the compactness decreased and reached approximately 25.10 Å during the last 25 ns. In the case of the other complexes, i.e., fluoxastrobin, trifloxystrobin, and azoxystrobin, the average Rg value was reported to be 24.90 Å for each complex. In conclusion, the Rg results show that all of the fungicides are stably bound inside the protein’s active site and possess stably binding dynamics. **Figure 7** shows the graphical representation of each complex as a function of time.

### 3.11 Residual flexibility of the fungicide-bound complexes

**Figure 8:**
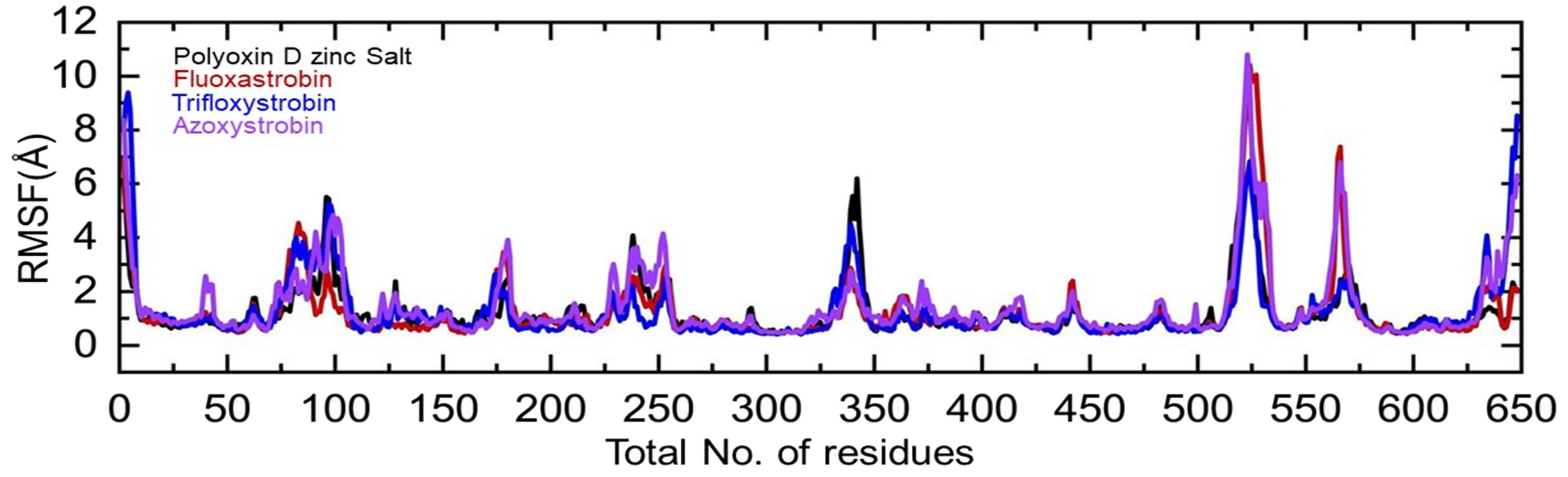
Residual flexibility of the top hits calculated as root mean square fluctuation. Each complex of polyoxin D zinc salt, fluoxastrobin, trifloxystrobin, and azoxystrobin is represented with a different color, respectively.

Residual flexibility plays an imperative role in determining the inhibitory effects, catalysis, and proteins’ conformational switches. Herein the residual flexibility was calculated as root mean square fluctuation (RMSF). The graph given in **Figure 8** shows that those regions 75–100, 225–250, 325–350, 500–540, and 555–575 are dynamically more flexible than the other regions. It can also be seen that the binding cavity regions, i.e., 101–175, 251–324, and 355–500, exhibit a lower dynamic fluctuation, which is reduced by the binding of each fungicide. This shows that the binding has affected the residual dynamics by inducing the inhibitory effects. The RMSF graph presenting each complex with different colors is shown in **Figure 8**.

### 3.12 Hydrogen bond count

**Figure 9:**
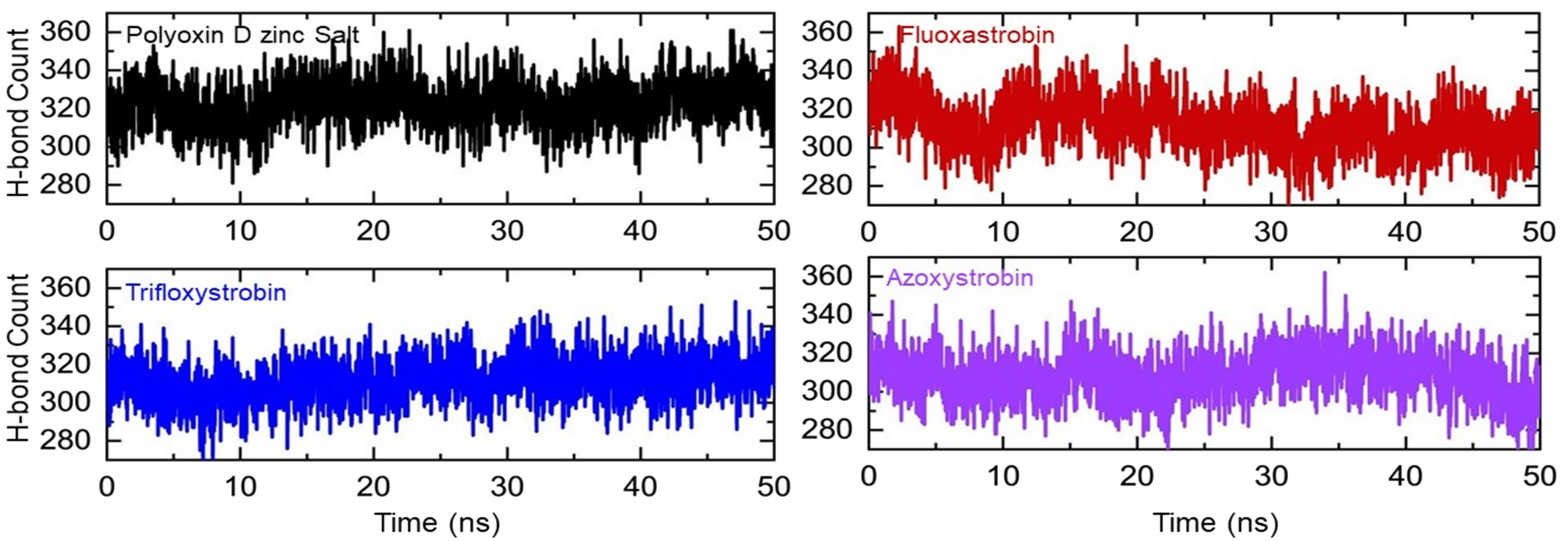
The total number of hydrogen bonds in each complex of polyoxin D zinc salt, fluoxastrobin, trifloxystrobin, and azoxystrobin is represented with a different color, respectively.

To evaluate the binding efficiency of each fungicide, the total number of hydrogen bonds is the best alternative estimate to reveal the binding robustness. The total number of hydrogen bonds during the simulation varies from frame to frame and provides a better view of the binding differences. Using the simulation time as a function of time, herein the total average number of bonds in the polyoxin D zinc salt complex was observed to be 320, while in fluoxastrobin during the first 10 ns the average number of bonds was more than that in polyoxin D zinc salt; however, the bonds are then reduced, and the average number of bonds was observed to be 310, which was similar in other complexes, i.e., trifloxystrobin and azoxystrobin. In azoxystrobin, the number of bonds gradually decreased after 45 ns and thus specified the unbinding events that occurred during the simulation. This shows that all of these fungicides exhibit a strong interaction cloud for the interactions and inhibition of glutamate–cysteine ligase protein. The hydrogen bond graphs are shown in **Figure 9**.

### 3.13 Binding free energy calculations

To connect the dynamic properties with the real-time binding free energy calculations and reveal the nearly experimental affinity of the top hits’ fungicides, molecular mechanics/generalized Born surface area (MM/GBSA) approach was employed. The total binding free energy for polyoxin D zinc salt was reported to be -41.76 kcal/mol; for fluoxastrobin, it was -39.06 kcal/mol; the TBE for trifloxystrobin was -33.77 kcal/mol, and for azoxystrobin, the total binding energy was -35.75 kcal/mol. This shows that these fungicides bind more robustly and block the GDP protein of *Taphrina deformans*. The other factors such as van der Waal energy (vdW) and electrostatic generalized Born (EGB) are also given in **Table 4**.

**Table 4:** Binding free energy of the top hits fungicides.

Complex	vdW	Electrostatic	EGB	Total binding energy
Polyoxin D zinc salt	-43.04	402.59	-394.97	-41.76
Fluoxastrobin	-50.30	-2.28	19.31	-39.06
Trifloxystrobin	-42.23	-5.00	19.42	-33.77
Azoxystrobin	-44.22	-5.35	19.75	-35.75

vdW, van der Waals interaction energy; EGB, electrostatic generalized Born energy.

All energies are given in kilocalorie per mole.

## 4 Discussion

Plant fungal pathogens are evolving and emerging new strategies to cause infections in plants around the globe (Zeng et al., 2018). This study addresses the growing need for novel antifungal targets due to the resistance developed by pathogens like *Taphrina deformans* against commonly used fungicides. The extensive use of these chemicals not only contributes to resistance but also poses environmental risks, underscoring the urgency for sustainable alternatives (Pecknold, 2015a). Due to the extensive and long-term use of fungicides, the pathogens developed resistance against widely used fungicides (Feng et al., 2020). To understand fungal pathogenesis and its control, it is important to study the genome and proteome of pathogens (Sammut et al., 2008). Our research utilized genomic and proteomic data to pinpoint essential genes in *T. deformans* that are promising drug targets because they show little homology with the host, *Prunus persica* (Luo et al., 2014; Sammut et al., 2008). Out of the whole genome, essential genes of pathogens that do not have a significant homology with the host can serve as potential drug targets (Luo et al., 2014). As the fungal infections’ incidence is increasing gradually, the demand for new antifungal drugs is also increasing. Hence, the key steps in developing a new drug are target identification and validation. To implement bioinformatics tools, it helps to identify, select, and prioritize potential drug targets (Hughes et al., 2011; Yang et al., 2012).

Oxidative stress responses have a crucial role in the virulence and survival of pathogens (Huynh et al., 2003; Mukherjee et al., 2009). Glutathione plays a key role in redox homeostasis and in cellular response to oxidative stress (Sipos et al., 2002). The critical enzyme involved in glutathione biosynthesis is glutamate–cysteine ligase (GCL). GCL is a cytoplasmic ligase considered as an essential protein for the survival and pathogenesis of *T. deformans*. In this study, we identified a protein, GCL of *T. deformans*, as a suitable antifungal target by subtractive proteomics approach. This finding is supported by our subtractive proteomics analysis, which also aligns with the methodologies used in previous studies to identify potential drug targets in pathogenic fungi(González-Fernandez et al., 2010; Bencurova et al., 2018; Jha et al., 2020; Shahid et al., 2020). The GCL 3D structure was modeled by comparative modeling that shows 90% confidence and 97% sequence identity with glutamate–cysteine ligase of *Saccharomyces cerevisiae* as shown in **Figure 4**. Comparative modeling is the most successful and accurate method to identify the structure of evolutionarily related proteins (Errami et al., 2003; Choong et al., 2011). However, in the previous literature, we could not find any data that shows GCL as an antifungal target. Furthermore, GCL was studied for active site identification, docking, molecular dynamic simulations, and free energy binding calculations against antifungal compounds.

The active sites of GCL were identified as Glu50, Glu110, Tyr111, His195, Gln272, Ser427, Trp430, Arg434, and Arg451 residues (**Figure 4**). In a structure-based drug discovery, the identification of active sites on a target protein has a great importance (Harigua-Souiai et al., 2015). The fungicides that interact by blocking the active sites have a significant effect on inhibiting the function of proteins (Aamir et al., 2018).

In this study, docking and simulation of GCL with fungicides were performed to find out the top hits by induced fit docking protocol, with those that scored greater than -7.50 kcal/mol selected as the best candidates (**Table 2**). The efficacy of fungicides like polyoxin D, fluoxastrobin, trifloxystrobin, and azoxystrobin was assessed through rigorous computational analyses, which showed a high binding affinity of these chemicals toward GCL, indicating their potential as effective fungicides. These fungicides were selected for their role in inhibiting vital functions within the pathogen, analogous to how azoxystrobin impedes mitochondrial respiration. Among the fungicide candidates, poloxin D’s score was the highest (-7.88 kcal/mol), and that of azoxystrobin was the lowest (-7.59 kcal/mol) as shown in **Table 2**. Polyoxin D is an antibiotic that is involved in cell wall synthesis inhibition as reported earlier (Endo et al., 1970). It has been studied that the use of polyoxin D against *Botrytis cinerea*, causal agent of gray mold on strawberry, has effective results against it (Dowling et al., 2016). Azoxystrobin inhibits mitochondrial respiration as it blocks the movement of electrons in the mitochondrial bc1 complex for mitochondrial electrons (Herms et al., 2002; Lu et al., 2019). The family of strobilurin fungicides is widely used around the world to combat white mold, rot, early and late leaf spot, rusts, and rice blast (Feng et al., 2020).

The MD simulations and free energy calculation of antifungal compounds polyoxin D zinc salt, fluoxastrobin, trifloxystrobin, and azoxystrobin with GCL showed that all of the complexes were stable at 50-ns simulation time (**Figure 6**). The average root mean square deviation for polyoxin D zinc salt was 2.0 Å, while for fluoxastrobin, trifloxystrobin, and azoxystrobin it was 3.0 Å. This shows that polyoxin D has a higher binding affinity to GCL than the other compounds. It has been reported in the previous study that the use of fungicides against *Fusarium* wilt, the two fungicides (oxathiapiprolin and famoxadone) with a potential energy of −113,166.16 and −112,628.96 kcal/mol, respectively, was revealed best via molecular docking and virtual screening. The stability of the protein–fungicide docked complexes was measured at 50-ns MD simulations. The average RMSD for the oxathiapiprolin was 2.49 and 2.42 Å, while for famoxadone it was 2.83 and 1.20 Å, respectively (Laskowski et al., 1993).

We evaluated the compactness as the radius of gyration (Rg) of each fungicide with GCL as given in **Figure 7**. For the structural compactness of fungicides with GCL active sites, the average Rg value for polyoxin D zinc salt was observed as 24.80 Å, while for fluoxastrobin, trifloxystrobin, and azoxystrobin it was 24.90 Å. Rg is considered as one of the essential parameters to calculate the binding stability of complexes inside the cavity (Khan et al., 2021a). This shows that all of the four fungicides can bind strongly to GCL. The strong binding affinity of fungicides with GCL can be confirmed by total hydrogen binding and total binding free energy calculations.

The total number of hydrogen bonds in the polyoxin D zinc salt complex was *n* = 320, while for fluoxastrobin, trifloxystrobin, and azoxystrobin this was *n* = 310 (**Figure 8**). Hydrogen bonding is a crucial stabilizing factor in the formation of biological complexes (Khan et al., 2021b). Polyoxin D has the highest number of hydrogen bonds than the other fungicides and can bind more firmly to GCL. The total binding free energy for all four fungicides was performed by MM/GBSA calculation (Errami et al., 2003). TBE for polyoxin D zinc salt was -41.76 kcal/mol; for fluoxastrobin, it was -39.06 kcal/mol; for trifloxystrobin, it was -33.77 kcal/mol; and for azoxystrobin, the TBE was -35.75 kcal/mol (**Table 3**). The analysis above revealed that polyoxin D shows more compactness and binding affinity to GCL. The results obtained for binding energy calculations of the protein–ligand interactions through MM/GBSA calculation were reported to be more effective (Genheden and Ryde, 2015). In previous studies, the MM/GBSA approach has proved to be the most accurate and reliable in protein–ligand docked complexes (Singh and Warshel, 2010; Sun et al., 2014). Binding free energy simulations are increasingly used in industries and academia for drug discovery (Feng et al., 2020).

From our study, it has been understood that GCL plays an important role in the metabolic processes of *T. deformans*. Its blocking and inhibition could control peach leaf curl disease. Furthermore, this protein has a significant potential for enhanced utilization in structure-based drug design and catalysis. The data from this study could drive future experimentation to design novel antifungal drugs by targeting the GCL of *T. deformans*. This approach not only offers a pathway to develop more effective fungicides but also contributes to the broader goal of achieving environmentally sustainable disease management practices. By focusing on specific targets like GCL, which are not present in the host organism, we can mitigate the impact on non-target species and reduce environmental contamination.

## 5 Conclusions

In conclusion, using subtractive proteomics approach, we identified the glutamate–cysteine ligase of *T. deformans* for the first time as a suitable antifungal target. The critical role of GCL in essential metabolic pathways, particularly in glutathione biosynthesis, positions it as an ideal candidate for novel fungicide development. Moreover, to further explore GCL’s potential as a fungicide, we performed two-step virtual screening and MD simulation analysis. The fungicides, such as, polyoxin D zinc salt, fluoxastrobin, trifloxystrobin, and azoxystrobin, showed strong binding affinity toward the active site of GCL, suggesting that GCL may be the best antifungal target in *T. deformans*. These findings have far-reaching ramifications. By targeting GCL’s crucial role in *T. deformans* survival and pathogenicity, we open new paths toward creating more effective fungicides capable of counter-resistance posed by the long-term use of existing ones. Furthermore, GCL could even be further utilized as part of drug design using its well-defined active sites for structure-based drug design; the computational tools used here—virtual screening and molecular dynamics—proved to be highly efficient at identifying potential drug targets for validation.

Future research should prioritize the synthesis and experimental validation of GCL fungicide analogs as potential next-generation treatments to effectively combat peach leaf curl disease while being environmentally safer to address concerns over chemical fungicide use. Furthermore, a rigorous examination into using GCL as a target against other fungal pathogens beyond *T. deformans* may extend these findings and contribute to global efforts in managing fungal diseases in agriculture.

This research presents an innovative and promising strategy to combat *T. deformans* by targeting glutamate–cysteine ligase. Our insights here could form the basis of future innovations in antifungal treatments that offer sustainable long-term solutions to manage peach leaf curl disease or other fungal infections.

## Data Availability

The original contributions presented in the study are included in the article/**Supplementary Material**. Further inquiries can be directed to the corresponding author.
