# Supplementary material for: Computational proteomics analysis of Taphrina deformans for the identification of antifungal drug targets and validation with commercial fungicides
Source: Front Plant Sci. 2024 Nov 7;15:1429890. doi: 10.3389/fpls.2024.1429890 (PMC11578757; doi:10.3389/fpls.2024.1429890)
Supplement: Supplementary file 1 [file DataSheet1.pdf]

**S1:** List of Bioinformatics tools used in the study

| TOOLS | ABBRVIATED FORM | WEB LINKS | REFERENCES |
|---|---|---|---|
| Universal protein Knowledge-base | UniprotkB | https://www.uniprot.org/ | Consortium. 2019. UniProt: a worldwide hub of protein knowledge. Nucleic Acids Res, *47*(D1), D506-D515. |
| Cluster Database at High Identity with Tolerance | CD-HIT | https://github.com/weizhongli/cdhit-web-server | Li, W., & Godzik, A. 2006. Cd-hit: a fast program for clustering and comparing large sets of protein or nucleotide sequences. Bioinform., 22(13), 1658-1659. https://doi.org/10.1093/bioinformatics/btl158. |
| Basic local alignment search tool protein | NCBI-BLASTp | https://blast.ncbi.nlm.nih.gov/Blast.cgi?PAGE=Proteins | Gul, H., Ali, S. S., Saleem, S., Khan, S., Khan, J., Wadood, A., ... & Wei, D. Q. 2020. Subtractive proteomics and immunoinformatics approaches to explore *Bartonella bacilliformis* proteome (virulence factors) to design B and T cell multi-epitope subunit vaccine. Infect. Genet. Evol, *85*, 104551 |
| Subcellular loacalization predictor 2 Gene ontology | CELLO2GO | (http://cello.life.nctu.edu.tw/cello2go/) | Yu, C. S., Chen, Y. C., Lu, C. H., & Hwang, J. K. 2006. Prediction of protein subcellular localization. Proteins., *64*(3), 643-651. https://doi.org/10.1002/prot.21018. |
| (KEGG Automatic Annotation Server) | KAAS | https://www.genome.jp/kegg/kaas/ | Moriya, Y., Itoh, M., Okuda, S., Yoshizawa, A. C., & Kanehisa, M. 2007. KAAS: an automatic genome annotation and pathway reconstruction server. Nucleic. Acids. *Res.*, *35*(suppl_2), W182-W185 |
| Kyoto encyclopedia of genes & genome | KEGG | https://www.genome.jp/kegg/kaas/ | Moriya, Y., Itoh, M., Okuda, S., Yoshizawa, A. C., & Kanehisa, M. 2007. KAAS: an automatic genome annotation and pathway reconstruction server. Nucleic. Acids. *Res.*, *35*(suppl_2), W182-W185 |
| Protein homology recognition engine 2 | Phyre 2 server | http://www.sbg.bio.ic.ac.uk/phyre2/html/help.cgi?id=help/faq | Kelley, L. A., Mezulis, S., Yates, C. M., Wass, M. N., & Sternberg, M. J. 2015. The Phyre2 web portal for protein modeling, prediction and analysis. Nat. Protoc., *10*(6), 845-858. https://doi.org/10.1038/nprot.2015.053. |
| Ramchandran plot analysis | Pro check server | https://saves.mbi.ucla.edu/ | Sheik, S. S., Sundararajan, P., Hussain, A. S. Z., & Sekar, K. (2002). Ramachandran plot on the web. *Bioinformatics*, *18*(11), 1548-1549. |
| Protein structure analysis | ProSA | https://prosa.services.came.sbg.ac.at/prosa.php | Wiederstein, M., & Sippl, M. J. 2007. ProSA-web: interactive web service for the recognition of errors in three-dimensional structures of proteins. Nucleic. Acids. Res., *35*(suppl_2), W407-W410. |

| | | | |
|---|---|---|---|
| Automatic Docking visaual informatics and navigation assistant | Auto Dock Vina | https://autodock.scripps.edu/ | Ravindranath, P. A., Forli, S., Goodsell, D. S., Olson, A. J., and Sanner, M. F. (2015). 657 AutoDockFR: advances in protein-ligand docking with explicitly specified binding site 658 flexibility. PLoS Comput Biol 11, e1004586 |
| Automatic Docking flexible receptors | Auto dock FR | https://autodock.scripps.edu/ | Ravindranath, P. A., Forli, S., Goodsell, D. S., Olson, A. J., and Sanner, M. F. (2015). 657 AutoDockFR: advances in protein-ligand docking with explicitly specified binding site 658 flexibility. PLoS Comput Biol 11, e1004586 |
| Chemistry<br>Post processing trajectory | CPPTRAJ<br><br>PPTRAJ | https://amberhub.chpc.utah.edu/amber-hub/ | Roe, D. R., and Cheatham III, T. E. (2013). PTRAJ and CPPTRAJ: software for processing and 660 analysis of molecular dynamics trajectory data. J Chem Theory Comput 9, 3084–3095. 661 |
| Molecular mechanics Poisson-Boltzmann surface area | MMPBSA | https://valdes-tresanco-ms.github.io/gmx_MMPBSA/dev/ | Chen, F., Liu, H., Sun, H., Pan, P., Li, Y., Li, D., et al. (2016). Assessing the performance of the 546 MM/PBSA and MM/GBSA methods. 6. Capability to predict protein–protein binding free 547 energies and re-rank binding poses generated by protein–protein docking. Physical 548 Chemistry Chemical Physics 18, 22129–22139 |
| General amber force field<br>Force field small molecules & biological macromolecule | GAFF<br><br>Ff14SB | https://ambermd.org/ | Case, D. A., Cheatham III, T. E., Darden, T., Gohlke, H., Luo, R., Merz Jr, K. M., ... & Woods, R. J. 2005. The Amber biomolecular simulation programs. J. Comput. Chem., 26(16), 1668-1688. https://doi.org/10.1002/jcc.20290 |
